# On an EUV Atmospheric Simulation Chamber to Study the Photochemical Processes of Titan’s Atmosphere

**DOI:** 10.1038/s41598-020-66950-6

**Published:** 2020-06-19

**Authors:** Jérémy Bourgalais, Nathalie Carrasco, Ludovic Vettier, Thomas Gautier, Valérie Blanchet, Stéphane Petit, Dominique Descamps, Nikita Fedorov, Romain Delos, Jérôme Gaudin

**Affiliations:** 1 LATMOS-IPSL, Université Versailles St-Quentin, CNRS/INSU, Sorbonne Université, UPMC Univ. Paris 06, 11 boulevard d’Alembert, 78280 Guyancourt, France; 2 CELIA, Université de Bordeaux – CNRS – CEA, UMR5107, 351 Cours de la Libération, F33405 Talence, France

**Keywords:** Ultrafast lasers, Atmospheric chemistry

## Abstract

The *in situ* exploration of Titan’s atmosphere requires the development of laboratory experiments to understand the molecular growth pathways initiated by photochemistry in the upper layers of the atmosphere. Key species and dominant reaction pathways are used to feed chemical network models that reproduce the chemical and physical processes of this complex environment. Energetic UV photons initiate highly efficient chemistry by forming reactive species in the ionospheres of the satellite. We present here a laboratory experiment based on a new closed and removable photoreactor coupled here to an Extreme Ultraviolet (EUV) irradiation beam produced by the high-order harmonic generation of a femtosecond laser. This type of EUV stable source allow long-term irradiation experiments in which a plethora of individual reactions can take place. In order to demonstrate the validity of our approach, we irradiated for 7 hours at 89.2 nm, a gas mixture based on N_2_/CH_4_ (5%). Using only one wavelength, products of the reaction reveal an efficient photochemistry with the formation of large hydrocarbons but especially organic compounds rich in nitrogen similar to Titan. Among these nitrogen compounds, new species had never before been identified in the mass spectra obtained *in situ* in Titan’s atmosphere. Their production in this experiment, on the opposite, corroborates previous experimental measurements in the literature on the chemical composition of aerosol analogues produced in the laboratory. Diazo-compounds such as dimethyldiazene (C_2_H_6_N_2_), have been observed and are consistent with the large nitrogen incorporation observed by the aerosols collector pyrolysis instrument of the *Huygens* probe. This work represents an important step forward in the use of a closed cell chamber irradiated by the innovative EUV source for the generation of photochemical analogues of Titan aerosols. This approach allows to better constrain and understand the growth pathways of nitrogen incorporation into organic aerosols in Titan’s atmosphere.

## Introduction

Among the many objects of great interest in the solar system, Titan, Saturn’s largest moon, is unique as a proxy for the early Earth. Titan has a thick atmosphere containing significant amounts of nitrogen and carbon through its main components (N_2_ and CH_4_)^1–4^, surface liquid hydrocarbons lakes^5^ and hydrological activities^6^. Exposure of Titan’s uppermost atmospheric layer (>700 km) to external energy sources (mainly solar photons and electrons from the Saturnian magnetosphere) leads to ionization and dissociation of the most abundant molecular species. First, these processes trigger efficient photochemistry forming relatively small neutral (<100 amu) molecules (*e.g*. nitriles, hydrocarbons) for which most observed abundances are now reasonably well reproduced by photochemical models^7–16^. However the formation of heavier compounds is not yet well understood^17^. Those heavy molecules induce a progressive formation of more complex organic compounds (*e.g*. Polycyclic Aromatic Hydrocarbons, heterocycles with nitrogen) at lower altitudes (*ca*. 500 km)^16^ which are the basis for the formation of aerosols (sub-*μ*m particles)^8^ which make up the orange-coloured photochemical hazes observed in the atmosphere of Titan. This complex chemical activity has been the target of several past (*Pioneer 11, Voyager I & II, Cassini-Huygens*)^18^ and future (*Dragonfly*)^19^ space missions on Titan and numerous laboratory experiments over the past two decades to reproduce different stages of its photochemistry. However, despite all the efforts to understand the molecular growth pathways that make Titan’s atmosphere so intriguing, questions regarding the formation of large molecules such as PAHs and complex organic particles remain unanswered, preventing a complete picture of the evolution of Titan from being obtained. For more details on Titan, readers can refer to recent reviews^20^.

Since *in situ* observations of Titan are insufficient to understand the chemistry of the atmosphere, recent decades have seen the growth of laboratory experiments on Titan’s atmospheric chemistry to simulate and analyze processes at work in the atmosphere. In order to reproduce the conditions observed on Titan, these devices use any type of discharge or radiation source (*e.g*. plasma discharge, UV irradiation) to simulate the energy sources that trigger organic chemistry in Titan’s atmosphere: energetic particles (cosmic rays and solar wind) and solar photons^21^. Experimental energy sources used in the laboratory generate radicals and other activated species by irradiating, under various physical parameters (temperature, pressure), a mixture of gases generally composed of N_2_ and CH_4_ which represent the major compounds in the atmosphere of Titan. Among these experimental energy sources, plasma discharge is the most popular choice for producing complex organic particles in the laboratory because of the high efficiencies associated with this method^22,23^ while photochemical yields are several orders of magnitude lower^24–26^. However, in Titan’s atmosphere, this approach is not the most appropriate. Indeed the solar UV radiation and EUV (Extreme Ultra Violet) photons are the main drivers of photochemistry on Titan since they are responsible for the almost total interaction of N_2_ and CH_4_^27^.

So far the most commonly used photon sources for experimental laboratory simulations of Titan’s atmosphere are low-pressure mercury and deuterium lamps limited to producing photons of 115 and 254 nm with *ca*. 10^16^ photons s^−1^ cm^−2^ ^28–31^. With such EUV-UV  sources (<300 nm), only photodissociation of methane can be achieved although previous laboratory simulations have shown that the presence of nitrogen increases the complexity of gas-phase and solid-phase chemistry and that nitrogen plays a key role in increasing the efficiency of gas-to-particle conversion^32–34^. Thus, the interesting range of photons that couple the chemistry of methane and nitrogen lies between the dissociation energy of methane and the ionization threshold of molecular nitrogen (79.4 nm *ca*. 15.6 eV), where N_2_ dissociates leading to both ground state and electronic excited states atomic N-fragments for which their role is still largely unknown and still to explore. So far, synchrotron sources are the most suitable to provide a high tunability in the narrow range of interest (98–79 nm) to couple methane and nitrogen chemistry in the context of photochemistry of Titan’s atmosphere^35–38^. Their unique disadvantage is the restricted access (*ca*. only few days/year) that by limiting the duration for the experimental campaigns reduces the physical multi-parametric studies. An alternative to synchrotron radiation is the high harmonic generation (HHG) of femtosecond laser^39,40^. These light sources deliver typically 200 meV broadband pulses at fixed wavelengths corresponding to odd harmonics of the fundamental laser. This fundamental wavelength can be tuned^41^ to get large coarse tunability. This table-top source emits from the EUV down to the water window spectral range^42^, depending on the generation gas chosen and the laser parameters. Combined with monochromator, selective dielectric mirror, or either metallic filter as used in the present work, such monochromatic EUV emission can be used as a possible, and more accessible, alternative to synchrotron radiation sources.

Synchrotron and HGG laser sources provide a sufficiently intense photon flux in the EUV range (*ca*. 10^10^–10^14^ photons s^−1^ cm^−2^) to reproduce the solar flux received by Titan over a realistic lapse of time for laboratory simulations. One day on Titan is equivalent to 15 days on Earth, so if we consider an average solar flux on Titan of *ca*. 5 × 10^6^ photons s^−1^ cm^−2^, a laboratory experiment will require a photon flux of *ca*. 2 × 10^9^ photons s^−1^ cm^−2^ for one hour to simulate one day on Titan. However, for a relevant gas mixture of Titan irradiated in the laboratory, the most crucial environmental factor in reproducing the chemistry of Titan’s atmosphere is the pressure that determines the molecular processes involved (photolysis, bimolecular or 3-body reactions). Indeed, the low pressure of Titan’s upper atmospheric layers (*ca*. <10^−6^ mbar) and the low rate of energy input mean that, on characteristic dynamical time scales, only a small fraction of the chemical bonds of the main atmospheric constituents are broken. Laboratory experiments must respect this low dose per reactive molecule by evaluating the exposure time in order not to use power densities that are too high compared to those actually observed in Titan’s atmosphere. Secondary photolysis processes of the photoproducts may occur and lead to the formation of unwanted species and overpolymerization compared to the initial aerosol formation processes in Titan’s atmosphere^36,43,44^. Experiments can then last up to hundreds of hours to form solid particles and generate enough material for *ex situ* analysis^29–31,45–47^.

Conducting experiments over a relatively long period of time leads to a number of other challenges in order to both get as close as possible to Titan’s atmospheric conditions and to limit contamination problems that can compromise the results during the generation of aerosol analogues. Thus, long experiments to irradiate gas mixtures of interest to Titan’s atmosphere with low EUV dose in the appropriate spectral range at low pressure to produce photochemical aerosols is a difficult task that has never been achieved before. It is then necessary to have a closed experimental device with a very low level of contamination and an analytical detection that minimizes destructive interferences to obtain a sufficient production of organic materials. To meet this challenge, we have developed an atmospheric simulation chamber, SURFACAT (french acronym for *SURFAtron Chambre A Tholins*), to study the photochemical processes in Titan’s atmosphere. As photon input window, we used an indium filter that allows EUV photons to pass through, but also closes our reactor. In this article, we present the SURFACAT atmospheric simulation chamber with its operating parameters, demonstrating its potential to reproduce chemistry in conditions as close as possible to Titan’s atmosphere. The analysis technique coupling a cryogenic trap with mass spectrometry is also presented. The EUV irradiation was produced from the femtosecond Ti:sapphire laser, AURORE, at CELIA laboratory in Bordeaux (France). We demonstrate here the feasibility to irradiate a gas mixture confined in SURFACAT by a stable EUV beam for several hours, with a photon wavelength of 89.2 nm, obtained from the 9th harmonic H9 of AURORE centred at 800 nm. We present first encouraging results on photoproducts detected during N_2_/CH_4_ (5%) gaseous mixture irradiation and demonstrating a coupling between carbon and nitrogen chemistry.

## Results

**Figure 1:**
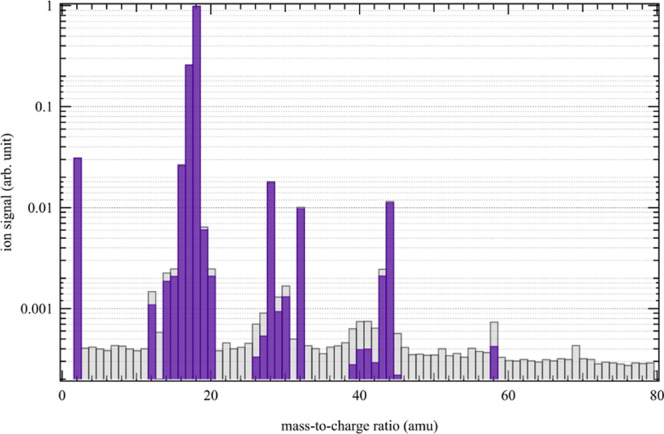
Mass spectrum with (purple bars) and without (grey bars) background subtraction obtained after 7 hours of trapping time at 89.2 nm.

The molecular species from the first photochemical experiments mimicking Titan’s upper atmosphere chemistry using an EUV-HHG beamline are shown in Fig. 1. The reactive gas mixture in the closed cell was irradiated for 7 hours at 89.2 nm with a measured photon flux of about *ca*. 2 × 10^10^ photons s^−1^ cm^−2^ after the 210 nm-thick indium membrane. The mass spectrum was recorded at the end of the irradiation after the release of the condensed photoproducts from the cryogenic trap (see Methods). The experiment has been performed twice providing equivalent results within a few %, similar to the measurement uncertainty of the mass spectrometer, demonstrating the reproducibility of the experiment. After subtraction of the background, the mass spectrum displayed in Fig. 1 in the mass range 2 to 80 amu shows no significant signal at high masses (>m/z 60). This is due to the fact that the residual gas analyser (RGA) mass spectrometer uses an electron gun to ionize the neutral molecules formed in the reactor . The electron beam with a typical energy of 70 eV causes fragmentation of the newly formed species, whose fragments contribute to the signals of masses lighter than the mass of the initial parent molecules. The mass spectrum is therefore the sum of the different fragmentation patterns (FPs) of the molecules in the reactor and its analysis is a real challenge due to the myriad of FPs.

It is then necessary to deconvolute the mass spectrum in order to identify and quantify the newly formed species. However, to overcome the multitude of possible solutions of mass spectrum deconvolution, we have introduced a Monte-Carlo type approach in our algorithm (see Methods). The database in Table of the Supplementary Information 1 (SI1) lists the species used for deconvolution with their respective FPs obtained from the NIST database. The 54 species used in our deconvolution were chosen based on our knowledge of the expected chemistry in a mixture of N_2_ and CH_4_ at 89.2 nm, with possible traces of water (see Methods). On this initial database, the relative intensities of the fragments of each molecule were varied by ±30% randomly to create *n* = 1 million new databases. Each of these databases was then tested on its ability to deconvolve the mass spectrum over the range from *m*/*z* 25 to *m*/*z* 60. The part of the spectrum below *m*/*z* 25 is not considered as a relevant criterion since most molecules fragment to these low masses. Of these one million deconvolutions, only the best 5% corresponding to the minimum residuals are retained as statistical solutions (see Fig. S1 in SI2). This set of statistical solutions is used to obtain the relative abundances of the species in the initial database by multiplying the relative intensity of the peaks by its electronic impact ionization cross section at 70 eV. This deconvolution set gives solutions for 30 species in the initial database. However, these species are not systematically found in each deconvolution and only those species whose mixing ratio is determined in a sufficiently large number of deconvolutions (>5% of the solutions) are kept here. The final list with 23 compounds present in the mass spectrum in Fig. 1 along with their recovered mixing ratios and standard deviation (1*σ*) are listed in Table 1. The probability distribution function of each of the compounds in Table 1 is given in Fig. S2 of the SI2. When distributions are not centred on a single mode, this indicates uncertainty about the formation of the molecule in the reactor. Uncertainties in the estimated mixing ratios, which may be high in some cases, are due to the fact that many fragments may be present in the mass of a species (see *eg*. ethylene at *m/z* 28 for example). However, although some species are found in only a small number of cases, their distribution may be found to converge to a single mode, increasing the probability of their presence in the reactor.

**Table 1:** List of compounds present in the mass spectrum with their retrieved mixing ratios and standard deviation (1*σ*).

Species	Formula	Mixing Ratio (ppm)	Standard Deviation (%)
butane	C_4_H_10_	9.9	40
acetone	(CH_3_)_2_CO	0.8	255
formamide	CH_3_NO	4.6	19
carbon dioxide	CO_2_	67	27
acetaldehyde	C_2_H_4_O	2.0	118
nitrous oxide	N_2_O	45	26
cyanamide	HNCNH	2.0	61
ketene	CH_2_CO	0.2	334
diazomethane	H_2_CNN	0.7	271
acetonitrile	CH_3_CN	4.7	36
propyne	CH_3_C_2_H	3.2	47
oxygen	O_2_	102	18
methanol	CH_3_OH	4.0	48
formaldehyde	H_2_CO	0.9	160
hydrogen cyanide	HCN	1.1	134
acetylene	C_2_H_2_	5.3	29
carbon monoxide	CO	163	18
methylamine	CH_3_NH_2_	1.4	185
methyl vinylacetylene	C_5_H_6_	1.6	135
ethyl vinylacetylene	C_6_H_8_	0.1	130
ethylene	C_2_H_4_	1.6	143
dimethyldiazene	CH_3_NNCH_3_	17	20
-propanamine	(CH_3_)_2_CHNH_2_	0.9	259

**Figure 2:**
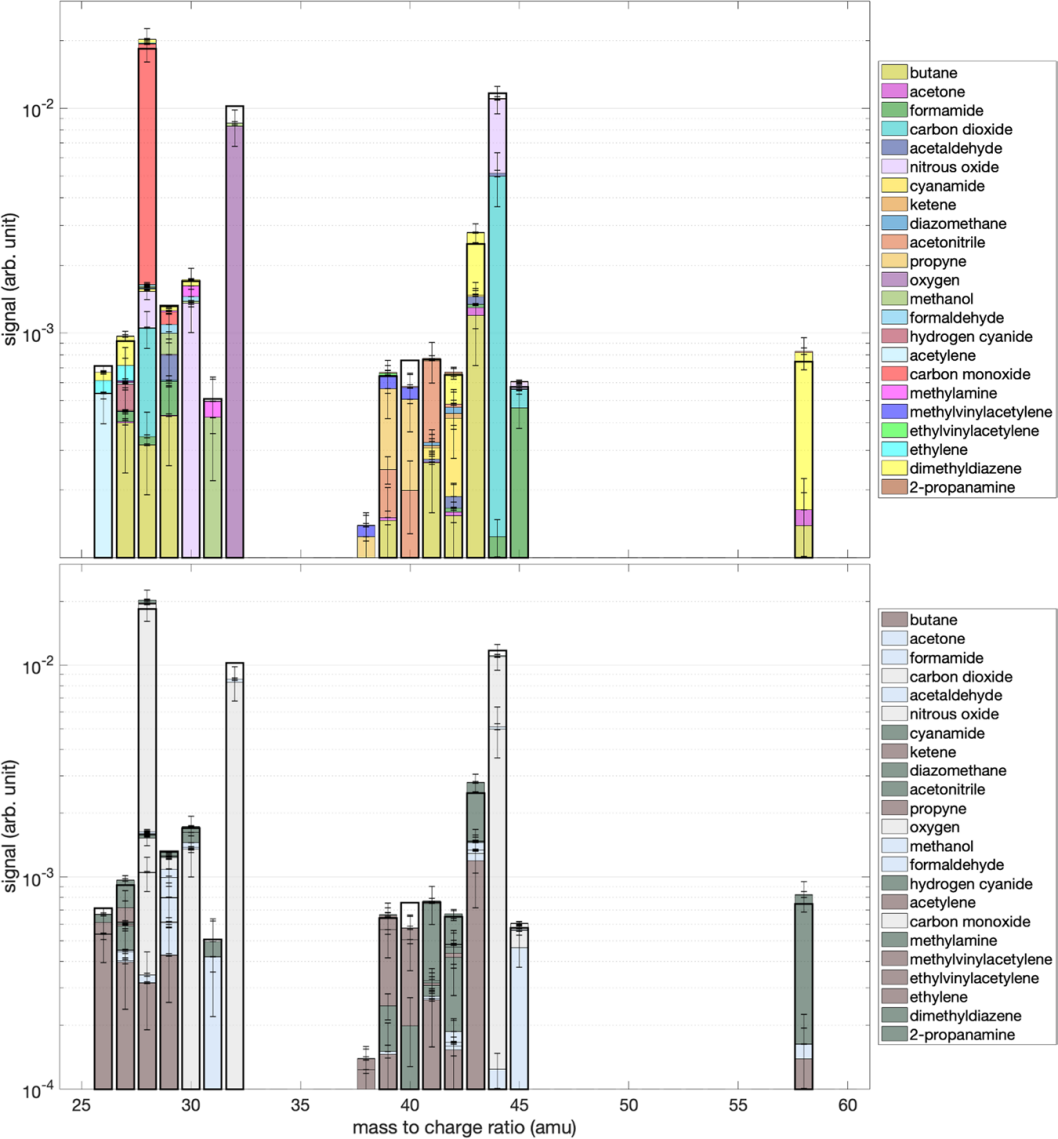
(upper panel) Decomposition of the mass spectrum shown in Fig. 1 using the Monte Carlo approach of Gautier *et al*.^64^. The experimental mass spectrum used for the decomposition is indicated by the black bold lines. The coloured bars indicate the contribution of each molecule. (lower panel) The coloured bars indicate the contribution of different families of chemical compounds: pollutants derived from water photolysis (grey), oxygenates (blue), hydrocarbons (brown) and nitrogen species (green).

The result of the deconvolution is displayed in Fig. 2 (upper panel) with the experimental mass spectrum (black line) and the calculated relative contribution of each species (colored bars). As shown in Fig. 2 (upper panel), our algorithm is able to reconstruct the mass spectrum, taking into account the relative uncertainties. The aim of this discussion is not to be exhaustive on all detected mass peaks, but to highlight the main neutral photoproducts and compare them to Titan’s photochemistry.

### Primary photochemical reactions and photoproducts

At this wavelength of 89.2 nm, the major products of CH_4_ photolysis are methyl (CH_3_), methylene (CH_2_) and methylidyne (CH) radicals and ionized methane (${{\rm{CH}}}_{4}^{+}$) (see Reactions 1)^48^.

1 $$\begin{array}{rcl}C{H}_{4}+h\nu \mathrm{(89.2}\,nm) & \to  & C{H}_{4}^{+}+{e}^{-}\\  & \to  & C{H}_{3}+H\\  & \to  & C{H}_{2}+H+H\\  & \to  & CH+{H}_{2}+H\end{array}$$

At the same time, at the wavelength used in this work the photolysis of molecular nitrogen leads to the formation of atomic nitrogen in both its ground state N(^4^S) and excited N(^2^D) (see Reaction 2)^14,48^.

2 $${N}_{2}+h\nu (89.2\,nm)\to N{(}^{2}D)+N{(}^{4}S)$$

Following these primary photochemical reactions, the reactivity of these radicals and ions will be at the origin of molecular growth by interaction with the most abundant molecules such as CH_4_ which forms ethylene (C_2_H_4_), the first hydrocarbon formed in this photochemical environment (see Reaction 3)^49^.

3 $$CH+C{H}_{4}\to {C}_{2}{H}_{4}+H$$

In the subsections that follow, we discuss the main pathways of species formation that are found by deconvolution of the mass spectrum in order to demonstrate the veracity of the statistical solutions.

### Pollutants and oxygenated species

A detailed examination of the major molecules obtained by the deconvolution process reveals that the dominant signals at *m/z* 28, 32 and 44 in the experimental mass spectrum are attributed mainly to carbon monoxide CO, oxygen O_2_, nitrous oxide N_2_O and carbon dioxide CO_2_ respectively. These species are the result of primary reactions that follow the photolysis of water vapour, the residual trace pollutant in the reactor, but they also come from the inevitable slow accumulation of air in the closed cell during the 7 hours by the micro-leaks of the reactor. At 89.2 nm, the photolysis of the water has three main exit channels, where the water can be ionized (H_2_O^+^) or dissociated to form the hydroxyl radical (OH) and the excited state oxygen atom O(^1^D) (see Reactions 4)^48^.

4 $$\begin{array}{rcl}{H}_{2}O+h\nu (89.2\,nm) & \to  & {H}_{2}{O}^{+}+{e}^{-}\\  & \to  & OH+H\\  & \to  & {H}_{2}+O{(}^{1}D)\end{array}$$

These photoproducts of water photolysis are very reactive and will collide mainly with the electrons resulting from the ionization of the species, the N_2_ and CH_4_ molecules of the initial gas mixture as well as their respective photoproducts resulting from their photolysis. The oxygen atom in its excited state will be quenched by nitrogen to be returned to its fundamental state O(^3^P). Subsequently, O(^3^P) by reaction with the methyl radical (CH_3_), leads to the formation of CO, a precursor for the formation of carbon dioxide (CO_2_) by reaction with the hydroxyl radical (OH) (see Reactions 5)^50,51^.

5 $$\begin{array}{rcl}O{(}^{3}P)+C{H}_{3} & \to  & CO+{H}_{2}+H\\ OH+CO & \to  & C{O}_{2}+H\end{array}$$

On the other hand, no training pathway at this wavelength and in this environment is conducive to a significant formation of O_2_. However, the reactor resides in a room that is at atmospheric pressure and the relatively low pressure of the reactor can lead to an accumulation of O_2_ through the micro-leaks of the reactor that can become significant after a long irradiation time.

Finally, N-atoms will subsequently react with water and OH photoproduct of its photolysis to form imidogen (NH) and nitric oxide (NO) radicals, which by recombining form nitrous oxide (N_2_O) (see Reactions 6)^52–54^.

6 $$\begin{array}{rcl}N{(}^{4}S)+OH & \to  & NO+H\\ N{(}^{2}D)+{H}_{2}O & \to  & NH+OH\\ NO+NH & \to  & {N}_{2}O+H\end{array}$$

The unwanted presence of oxygen in the reactor leads to partial oxidation of the organic compounds that form, but thanks to the selectivity of the experimental conditions and statistical analysis that is used, it is possible to easily estimate its impact. Methanol (CH_3_OH) and formaldehyde (H_2_CO) are oxygenated molecules that are easily formed from the products of the photolysis of water and methane. Formamide is the only molecule found in this work with one oxygen atom and one nitrogen atom. Its formation is also facilitated by the reactivity between the NO and CH_3_ radicals present in abundance in the reactor (see Reactions 7)^52,55^.

7 $$\begin{array}{rcl}OH+C{H}_{3} & \to  & C{H}_{3}OH\\ {H}_{2}O+CH & \to  & {H}_{2}CO+H\\ NO+C{H}_{3} & \to  & C{H}_{3}NO\end{array}$$

Two molecules with 2 carbon atoms were also found, acetaldehyde (CH_3_CHO) and ketene (CH_2_CO). CH_2_CO comes from the reaction between H_2_CO with the methylidyne radical (CH) coming from the photolysis of methane. The formation of CH_3_CHO requires the formation of the intermediate HCO via the reaction between O(^3^P) with the methylene radical (^3^CH_2_). HCO then reacting with CH_3_ leads to the formation of CH_3_CHO (see Reactions 8)^51,52^.

8 $$\begin{array}{rcl}{H}_{2}CO+CH & \to  & C{H}_{2}CO+H\\ O{(}^{3}P)+{}^{3}C{H}_{2} & \to  & HCO+H\\ HCO+C{H}_{3} & \to  & C{H}_{3}CHO\end{array}$$

Finally, acetone ((CH_3_)_2_CO) is the most complex molecule found with 3 carbon atoms. (CH_3_)_2_CO comes from the reaction between the acetyl radical (CH_3_CO) with CH_3_. This intermediate is formed via the reaction between O(^3^P) with C_2_H_4_ (see Reactions 9)^51,52^.

9 $$\begin{array}{rcl}O{(}^{3}P)+{C}_{2}{H}_{4} & \to  & C{H}_{3}CO+H\\ C{H}_{3}CO+C{H}_{3} & \to  & {(C{H}_{3})}_{2}CO\end{array}$$

More important for the photochemistry of Titan, these oxygen species do not act as chemical intermediates in the formation of oxygen-free products. Rather, they will consume hydrocarbon radicals and nitrogen atoms as shown previously and thus reduce the formation of complex organic species. However, in spite of the formation of these undesirable oxygen species, more than 60% of the other retrieved species are O-free hydrocarbons and nitrogen species (see low panel in Fig. 2).

### Hydrocarbons

Several hydrocarbons were recovered, ranging from small hydrocarbons with two carbon atoms to one hydrocarbon containing six carbon atoms. The small hydrocarbons are acetylene (C_2_H_2_) and ethylene (C_2_H_4_). As mentioned in the previous section, C_2_H_4_ is one of the primary photoproducts of photochemical environments containing N_2_ and CH_4_ (see Reaction 9). Its very efficient formation pathway allows its successive photolysis by photons at 89.2 nm leading to several output pathways, one of which leads to C_2_H_2_ observed at *m/z* 26 (see Reactions 10)^48^.

10 $$\begin{array}{rcl}{C}_{2}{H}_{4}+h\nu (89.2\,nm) & \to  & {C}_{2}{H}_{4}^{+}+{e}^{-}\\  & \to  & {C}_{2}{H}_{2}^{+}+{H}_{2}+{e}^{-}\\  & \to  & {C}_{2}{H}_{3}^{+}+H+{e}^{-}\\  & \to  & {C}_{2}{H}_{2}+H+H\\  & \to  & {C}_{2}{H}_{2}+{H}_{2}\end{array}$$

C_2_H_4_ is the key photoproduct of the hydrocarbon growth observed in the reactor. By reaction with the CH radical, it allows the formation of propyne (CH_3_C_2_H) and the different pathways of its photolysis are at the origin of the formation of the complex hydrocarbons that were found (see Reaction 11)^56,57^.

11 $$\begin{array}{ccc}{C}_{2}{H}_{4}+CH & \to  & C{H}_{3}{C}_{2}H+H\end{array}$$

Heavier ions (${{\rm{C}}}_{3}{{\rm{H}}}_{4}^{+}$, ${{\rm{C}}}_{3}{{\rm{H}}}_{5}^{+}$, ${{\rm{C}}}_{4}{{\rm{H}}}_{7}^{+}$) are formed during the photolysis of ethylene by reaction with the ethylene itself or methane by the formation of acetylene and ethylene ions. These heavier ions will then recombine with electrons and dissociate in order to form radicals with high masses (C_3_H, C_3_H_3_, C_3_H_7_) which will be able to react with the abundant radicals coming out like CH_3_ to form the complex hydrocarbons: butane (C_4_H_10_), methyl vinylacetylene (C_5_H_6_), and ethyl vinylacetylene (C_6_H_8_), which were found by statistical analysis (see Reactions 12)^51,52,56–58^.

12 $$\begin{array}{l}\begin{array}{rcl}{C}_{2}{H}_{4}^{+}+{C}_{2}{H}_{4} & \to  & {C}_{4}{H}_{7}^{+}+H\\ {C}_{2}{H}_{2}^{+}+C{H}_{4} & \to  & {C}_{3}{H}_{5}^{+}+H\\ {C}_{2}{H}_{2}^{+}+C{H}_{4} & \to  & {C}_{3}{H}_{4}^{+}+{H}_{2}\end{array}\\ \begin{array}{rcl}{C}_{4}{H}_{7}^{+}+{e}^{-} & \to  & {C}_{3}{H}_{7}+C\\ {C}_{3}{H}_{5}^{+}+{e}^{-} & \to  & {C}_{3}H+{H}_{2}+{H}_{2}\\ {C}_{3}{H}_{4}^{+}+{e}^{-} & \to  & {C}_{3}{H}_{3}+H\end{array}\\ \begin{array}{rcl}C{H}_{3}+{C}_{3}{H}_{7} & \to  & {{\bf{C}}}_{4}{{\bf{H}}}_{10}\\ C{H}_{3}+{C}_{3}H & \to  & {C}_{4}{H}_{3}+H\\ C{H}_{3}+{C}_{4}{H}_{3} & \to  & {{\bf{C}}}_{5}{{\bf{H}}}_{6}\\ C{H}_{2}+{C}_{2}{H}_{4} & \to  & {C}_{3}{H}_{5}+H\\ {C}_{3}{H}_{3}+{C}_{3}{H}_{5} & \to  & {{\bf{C}}}_{6}{{\bf{H}}}_{8}\end{array}\end{array}$$

### N-species

Several nitrogenous species were also found, containing one to two nitrogen atoms. The simplest is hydrogen cyanide (HCN), whose formation comes from the reaction between N(^4^S) with small radicals coming from the photolysis of CH_4_: CH_2_ and CH_3_ (see Reactions 13)^51,59^.

13 $$\begin{array}{rcl}N{(}^{4}S)+C{H}_{3} & \to  & HCN+H+H\\ N{(}^{4}S)+C{H}_{2} & \to  & HCN+H\end{array}$$

Another way out of the reaction between N(^4^S) and CH_3_ is the formation of the radical H_2_CN which by reaction with hydrogen atoms is also a minor source of HCN (see Reactions 14)^51,59^.

14 $$\begin{array}{rcl}N{(}^{4}S)+C{H}_{3} & \to  & {H}_{2}CN+H\\ {H}_{2}CN+H & \to  & HCN+H+H\end{array}$$

As for the nitrogen atoms N(^2^D), they allow methanimine (CH_2_NH) to form very quickly by reaction with CH_4_, which is not found in the deconvolution analysis because its fragmentation spectrum is not known and is therefore not present in the database. However CH_2_NH is undeniably formed in all photochemical environments with N_2_ and CH_4_ and is a major contributor to the growth of nitrogen chemistry in the reactor. By reaction with CH it forms the detected acetonitrile (CH_3_CN) but also by reaction with N(^2^D), the cyanamide (HNCNH) and diazomethane (H_2_CNN) isomers which contain two nitrogen atoms (see Reactions 15)^51^.

15 $$\begin{array}{rcl}N{(}^{2}D)+C{H}_{4} & \to  & C{H}_{2}NH+H\\ CH+C{H}_{2}NH & \to  & {\bf{C}}{{\bf{H}}}_{3}{\bf{CN}}+H\\ N{(}^{2}D)+C{H}_{2}NH & \to  & {\bf{HNCNH}}+H\\ N{(}^{2}D)+C{H}_{2}NH & \to  & {{\bf{H}}}_{2}{\bf{CNN}}+H\end{array}$$

These species, which witness molecular growth from nitrogen chemistry, are not formed via the oxygenated pollutants found and described in a previous section. However, oxygenated pollutants alter their relative abundance by taking up small hydrocarbon and nitrogen radicals. However, the presence of methylamine (CH_3_NH_2_) shows that we must be cautious in our analysis. Its formation is initiated by the reaction between CH_4_ and its ionized form ${{\rm{CH}}}_{4}^{+}$ giving the ion ${{\rm{CH}}}_{5}^{+}$. This ion will react with H_2_O to form the hydronium ion (H_3_O^+^) which in turn reacts with CH_2_NH to form the ${{\rm{CH}}}_{2}{{\rm{NH}}}_{2}^{+}$ ion. ${{\rm{CH}}}_{2}{{\rm{NH}}}_{2}^{+}$ only has to recombine with an electron to dissociate to form NH_2_ which once formed will react with CH_3_ to give CH_3_NH_2_ (see Reactions 16). The formation of NH_2_ requires several steps but which are efficient formation pathways, explaining the possible formation of CH_3_NH_2_ in the reactor despite the high uncertainty related to its regained mixing ratio. It is the only nitrogen species found whose formation would have been supported by the presence of oxygen species in the reactor^60–62^.

16 $$\begin{array}{rcl}C{H}_{4}^{+}+C{H}_{4} & \to  & C{H}_{5}^{+}+C{H}_{3}\\ C{H}_{5}^{+}+{H}_{2}O & \to  & {H}_{3}{O}^{+}+C{H}_{4}\\ {H}_{3}{O}^{+}+C{H}_{2}NH & \to  & C{H}_{2}N{H}_{2}^{+}+{H}_{2}O\\ C{H}_{2}N{H}_{2}^{+}+{e}^{-} & \to  & C{H}_{2}+N{H}_{2}\\ N{H}_{2}+C{H}_{3} & \to  & {\bf{C}}{{\bf{H}}}_{3}{\bf{N}}{{\bf{H}}}_{2}\end{array}$$

In contrast to hydrocarbons, there is a lack of data on the FPs of nitrogenous species, thus limiting the species assigned in this work. However, dimethyldiazene (CH_3_NNCH_3_) and 2-propanamine ((CH_3_)_2_CHNH_2_) has been identified as the heaviest nitrogenated species in this work. Their gas phase formation pathways are not known but these species have also been found in previous studies using an atmospheric plasma glow discharge in N_2_-CH_4_ gas mixtures^63^.

## Discussion

First encouraging results were obtained after 7 hours of irradiation at 89.2 nm with a flux estimated at about 2 × 10^10^ photons s^−1^ cm^−2^. In spite of the precautions taken, the mass spectrum analysis highlights the presence of oxygen species coming from the slow but inevitable micro-leaks with the closed cell reactor, which prevents us from being too affirmative in the results put forward in this paper. Despite this, complex species comprising several carbon and nitrogen atoms were found through extensive statistical analysis without any insertion of oxygen atoms^64^. Their formation can be explained simply by some reactions resulting from the photochemistry initiated by the photons with the initial gas mixture (N_2_ and CH_4_). These species will now be placed in the context of Titan’s atmospheric chemistry in order to highlight the interest of these first promising results.

The detection of heavy hydrocarbons with several carbon atoms demonstrates the feasibility of the reactor to initiate a molecular growth similar to that of Titan’s upper atmosphere despite the wavelength selectivity of this experiment^65^. Small molecules such as acetylene (C_2_H_2_) and ethylene (C_2_H_4_) support the experiment’s ability to trigger Titan’s complex chemical network from the photolysis of methane and nitrogen because they are abundant products in Titan’s chemistry^66^. C_2_H_4_ is formed in the upper atmosphere and diffuses downwards while being photolysed and is the main source of C_2_H_2_ in the bulk of the atmosphere. In Titan’s upper atmosphere, C_2_H_4_ is responsible for the growth of hydrocarbons with, in particular, the formation of propyne (CH_3_C_2_H) and allene (CH_2_CCH_2_) isomers. However, in our reactor the statistical analysis found only one of the two isomers although both FPs are present in the database. This absence comes from the fact that CH_2_CCH_2_ tends to be easily isomerized to CH_3_C_2_H. The abundance of CH_3_CCH_2_H is therefore self-sustaining while CH_2_CCH_2_ tends to disappear but both isomers are likely to be formed in the reactor. The fact that our statistical analysis finds only one of the two isomers, shows us its sensitivity to be able to discriminate the presence of molecule with the same raw formula once the respective FPs are known.

On the opposite, butane (C_4_H_10_) is formed via a different formation pathway than in Titan’s atmosphere. In Titan’s atmosphere, C_4_H_10_ is formed from propylene (C_3_H_6_) which itself is derived from C_2_H_6_^15^. However, the formation of C_2_H_6_ is not optimum at 89.2 nm, due to the fact that the main reaction involves two CH_3_ radicals. At 89.2 nm the photolysis branching ratios of CH_4_ favour the formation of ${{\rm{CH}}}_{4}^{+}$ and CH before that of CH_3_ . Consequently, not enough CH_3_ are produced to react together as it occurs on Titan at λ below 100nm. In the reactor the formation of C_4_H_10_ is via C_2_H_4_ which, by means of ion-molecule reactions and dissociative recombination, makes it possible to form the intermediate C_3_H_7_ necessary for the formation of C_4_H_10_. These reactions are much more efficient than the neutral pathways initially mentioned for the formation of C_4_H_10_.

Thus, despite wavelength selectivity, this work demonstrates that it is possible to form complex hydrocarbons similar to those found on Titan but via different formation pathways. The analysis also identified two even higher hydrocarbons methyl vinylacetylene (C_5_H_6_) and ethyl vinylacetylene (C_6_H_8_) which have not yet been officially detected in Titan’s atmosphere but have been found in thermal degradation studies of Titan aerosol analogues^67^. However, the detection of these species is to be taken with caution because their dominant peaks, which is above mass 60, does not appear. These molecules are formed via the dissociative recombination of heavy ions (${{\rm{C}}}_{3}{{\rm{H}}}_{4}^{+}$, ${{\rm{C}}}_{3}{{\rm{H}}}_{5}^{+}$) which form new hydrocarbon radicals (C_3_H, C_3_H_3_, C_3_H_5_, C_4_H_3_) that will react with the radicals coming from the photolysis of methane. This work proposes efficient pathways to form new heavy hydrocarbons whose respective masses are observed in the *Ion and Neutral Mass Spectrometer* (INMS) spectra of the *Cassini* probe but which remain unassigned^21^.

As far as the formation of nitrogen species is concerned, it is initiated by the dissociation of molecular nitrogen above 600 km in Titan and for wavelengths below 100 nm, like the one used in this work, which produce both atoms in their ground and excited state. A few nitrogenous species were detected in this work, including hydrogen cyanide (HCN) which is the smallest molecule observed and a very stable molecule well known from Titan’s atmospheric chemistry due to its C ≡ N triple bond that is difficult to break^68^. Its main route of formation in Titan and the reactor is via the reaction between nitrogen atoms and methylene-amidogen (H_2_CN) radicals and constitutes building blocks to build up more complex nitrile species through proton-transfer reactions with ions, whose associated neutrals have a lower proton affinity. Similarly, acetonitrile (CH_3_CN) once formed will protonate in Titan’s atmosphere and comes mainly from the reaction between N(^2^D) and C_2_H_4_. However in our reactor C_2_H_4_ is mostly photodissociated or ionized and it is likely that a larger source of CH_3_CN comes from the reaction between CH and methanimine (CH_2_NH), a reaction that is not mentioned in the photochemical models of Titan although it would be an efficient source of CH_3_CN. Although this product was not found in the reactor because its fragmentation pattern is not known, there is every reason to believe that it is present because of the detection of the two isomers: cyanamide (HNCNH) and diazomethane (H_2_CNN) which are the products of the reaction of CH_2_NH with nitrogen atoms. The detection of these two isomers is a very interesting result because current photochemical models, although taking into account nitriles to model nitrogen chemistry, only contain species with a single nitrogen atom. There are no diazo and triazo species taken into account although their involvement is necessary to explain the formation of observed nitrogen-rich aerosols in Titan’s atmosphere. Laboratory analysis of aerosol analogues identified nitrogen-rich aromatic species for which cyanamide was presented as a possible heteroaromatic structure to explain the observed N = N patterns along with other CN_2_H_2_ isomers^69–72^. Referring to the importance of anion chemistry on the growth of aerosols in the atmosphere of Titan^8^, recent experimental results have identified new negatively charged di- and tri-nitrogen species (${{\rm{CN}}}_{2}^{-}$, ${{\rm{CHN}}}_{2}^{-}$, ${{\rm{CH}}}_{2}{{\rm{N}}}_{2}^{-}$) in a plasma of N_2_/CH_4_, suggesting that a growth in nitrogen chemistry would pass through the anions^73^. The isomers of CH_2_N_2_ have been evoked as precursors to the formation of these anions via (dissociative) electronic attachment reactions. Notably the reaction involving HCN and ammonia (NH_3_) has been suggested as a route of formation to cyanamide. However, the presence of cyanamide in this work is unlikely because cyanamide is in the solid phase at room temperature. Therefore, if cyanamide had formed during the experiment, it would likely have deposited on the chamber wall as a solid and would not condense in the cold trap. Deconvolutions of the mass spectrum by removing cyanamide from the database show that only the relative abundances of ketene and diazomethane are affected. The results show an increase in the abundance of ketene at 1.1 ppm (1*σ* = 130%) and diazomethane at 3.5 ppm (1*σ* = 91%). It is therefore interesting to see that the results of the algorithm are robust and that the elimination of cyanamide reinforces the presence of a diazotized compound, as diazomethane. This work highlights here simple alternative formation pathways involving N(^2^D) and CH_2_NH, which has been experimentally proven in the past to form easily ^74^. These CH_2_N_2_ isomers should be taken into account in the photochemical models. This work supports the results of Dubois *et al*.^75^ and studies on the chemical composition of aerosol analogues, by showing that complex nitrogen species, with several nitrogen atoms, can form easily in photochemical environments containing N_2_ and CH_4_.

Finally the detection of dimethyldiazene (CH_3_NNCH_3_) and 2-propanamine ((CH_3_)_2_CHNH_2_) is consistent with the high nitrogen incorporation observed by the Huygens probe aerosol collector pyrolysis instrument, which identified NH_3_ and HCN as fingerprints of the chemical structure of the complex nitrogenated organic compounds that make up the aerosol nucleus.^76^. These are the largest masses produced in the present experiment after 7 hours of irradiation at 89.2 nm and are a very encouraging result on the path of formation of photochemical aerosol analogues in the laboratory. Once again the detection of  these molecules demonstrates with a single wavelength, the feasibility of producing complex molecules with several nitrogen atoms that are absent from Titan’s photochemical models.

In conclusion, our results highlight a photochemistry at 89.2 nm close to the atmospheric chemistry of Titan with large hydrocarbons especially nitrogen-rich organic compounds, up to 2 nitrogen atoms. Among these nitrogen compounds, new species that have not been observed *in situ* corroborate previous experimental measurements during laboratory simulations on similar gas mixtures and on the chemical composition of aerosol analogues. This work represents an important step in the use of a closed cell chamber for the generation of Titan-type photochemical aerosol analogues to better constrain the nitrogen fixation processes in Titan’s atmosphere and its relevance to the evolution of primitive life. This project is the first step in a long-term strategy to exploit various VUV sources. This paper highlights the potential of the HHG as a source of VUV for planetary atmospheric studies. Current limitations due to contamination from slow but unavoidable micro-leaks and residual water that introduce oxygen into the chemical system will require improvements such as the use of a higher EUV flux to reduce the irradiation time. This new type of high repetition rate EUV sources (above 200 kHz) has recently been optimised^41^, paving the way for shorter irradiation time and therefore oxygen-free experiments. The cold trap could also be used before performing the experiment in order to minimize the water signal in the chamber. Developments are also under way to house the reactor in a secondary containment with the possibility of heating the entire facility. This secondary containment would be filled with dry N_2_, to avoid micro-leaks that lead to the introduction of water and oxygen into the reactor, even over long irradiation periods.

## Methods

The experimental setup can be sub-divided in two main parts: the High Harmonic Generation (HHG) beamline, including generation and beam transport and the atmospheric chamber for photochemical tholins generation (SURFACAT) and diagnostics as depicted in Fig. 4 of the entire experimental setup.

### High-harmonic generation

**Figure 3:**
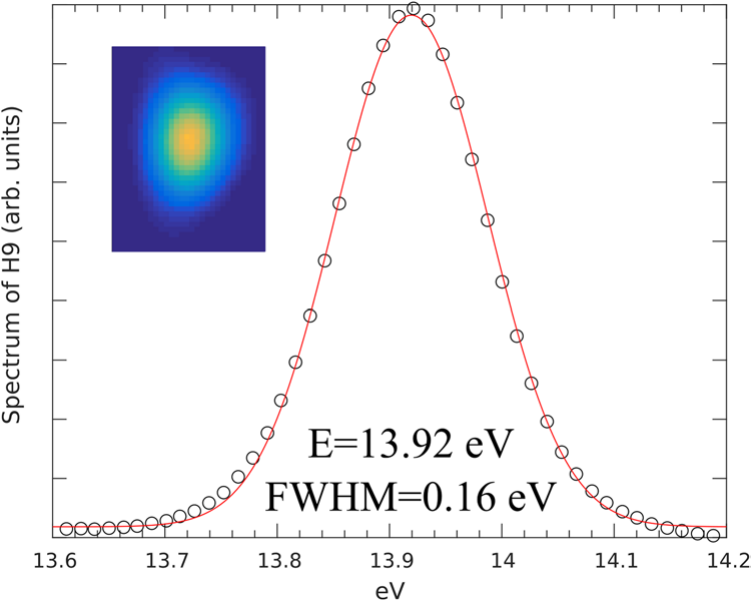
Spectrum of H9 centered at 89.2 nm recorded on the VUV spectrometer with its spatial vertical mode in inset (horizontal mode is the plane of dispersion of the grating).

We used the AURORE femtosecond Ti:sapphire laser at CELIA laboratory in Bordeaux (France) which is feeding up to 5 five beamlines dedicated to ultra-fast phenomena. The laser is delivering 7 mJ pulses of 30 fs FWHM duration at a repetition rate of 1 kHz. The spectrum is centered at a wavelength of 800 nm (h*v* = 1.55 eV) with a FWHM of 50 nm. The laser beam is focused by a plano-convex lens of 1.5 m focal in a gas jet backed by C_2_H_2_ gas. The typical backing pressure was 130 mbar, while the pressure in the generation chamber is rising up to 10^−2^ mbar. A differential pumping stage is located in between the generation chamber and the rejection chamber decreasing the pressure down to 7.2 × 10^−6^ mbar. The beam can be either steered to the SURFACAT reactor by reflection on a SiO_2_ mirror with a Nb_2_O_5_ anti-reflective coating for the 1.55 eV/800 nm (fundamental wavelength), or to the EUV spectrometer if the mirror is retracted. The reflectivity of this SiO_2_ mirror at 70° in the 89.2 nm range is maximized for a S polarization and has been measured to be 78.9%, while restraining the reflectivity of the 800 nm to 0.25%. These reflectivity values maintain the EUV flux without to melt the 210 nm-mesh supported indium filter with the impinging intense 800 nm pulse. The indium filter is the key optical element that both selects the 14 eV, defines the optical entrance of the reactor SURFACAT and maintains the volume/pressure of the reactants. The 89.2 nm radiation is characterized with the EUV spectrometer, made of entrance slit (1 mm aperture) and a variable line-space grating. The dispersed HHG beam as shown on Fig. 3 is then focused in the dispersion plane to a micro-channel plate which amplifies and converts the photon signal to an electron signal.

The electron signal is then converted back to visible photons signal which can be monitored by a CCD camera located outside the vacuum chamber. This spectrometer allows us to monitor the HHG to tune online the different relevant parameters to optimize the H9 photon flux. The full width half maximum spectral bandwidth of the H9 was measured to be 0.16 eV. As the beam is refocused only along the dispersion plane the size of the measured spot in the vertical direction corresponds to the beam size, allowing us to measure the beam divergence. This later one is measured to be 9 × 10^−2^ degree. The distance from the HHG source-indium filter is fixed such that the HHG beam diameter is equal to the clear aperture of the filter, *ie*. 15.9 mm. Once the optimization is achieved the rejection mirror is inserted in the beam.

### The atmospheric chamber (SURFACAT) coupled to a cryogenic trap

At the end of the light line, the EUV beam arrives at the indium membrane that isolates the SURFACAT chamber and allows it to be easily connected to various VUV light sources. The 210 nm thick indium filter that isolates the SURFACAT chamber from the beamline acts as a monochromator. Indium is the only material capable of being used as a spectral filter in the EUV wavelength range, particularly in the 75 nm to 100 nm range, as relatively few materials have transparency at these wavelengths^77,78^. Up to 120 nm, MgF_2_ and LiF optics are commonly used to focus or disperse VUV, but the transmission of these materials decreases at shorter wavelengths, falling to zero at 104 and 116 nm for LiF and MgF_2_, respectively^79^.

However, precautions must be taken to ensure that the transmission of indium filters does not vary during irradiation experiments. During these long photochemistry experiments, photons are absorbed very rapidly in a very small volume that constitutes the reactive zone, just behind the indium membrane. As the gas is not renewed in a closed cell, there can then be an accumulation of impurities on the surface of the indium membrane, resulting in a change in photon flux, thus reducing the chemistry present^80^. It is therefore vital to have a record of the stability of the photon flux that passes through the Indium membrane during irradiation experiments. This is why a 100 mm^2^ XUV photodiode (Opto Diode AXUV 100 G) retractable is positioned downstream of the indium membrane in order to make a measurement before and after irradiation to ensure that the membrane transmission has not changed. With a 1 kHz EUV source, the results show no change in membrane transmission even after several hours of irradiation. Theoretically, the membrane that was used in this work has a transmission of 1.7 × 10^−2^ (H7 = 10.8 eV), 0.21 (H9 = 89.2 nm) and 4.6 × 10^−3^ (H11 = 17.0 eV) (CXRO data http://henke.lbl.gov/optical_constants/). In practice, we have seen a transmission of 15% at 89.2 nm with a current measured by the photodiode of 8 nA, which corresponds to a flux of *ca*. 2 × 10^10^ photons s^−1^.

The behaviour of a photochemical system depends on the quantity of photons per reagent but also on the optical depth of the gas mixture in the cell. The optical depth of the gas mixture influences the photochemical processes^25,45,81^. The processes in a photon-dominated environment will not be the same as in a reactant-dominated environment. The higher the gas density, the shallower the depth at which photons enter the photochemical chamber. At shallow optical depths, photons penetrate deep and cause chemical reactions with primary photoproducts, increasing molecular diversity and ultimately aerosol production, while deep optical depths limit photons to primary chemical reactions only. However, Titan’s atmosphere has an optically thick atmosphere^11,27^ but it is therefore necessary to find a compromise in order to be able to produce organic matter in large enough quantities to carry out *ex situ* analyses. The optical depth in our reaction cell increases with increasing distance from the indium membrane (due to the increasing gas mixture column encountered) and is a function of initial gas mixture density and wavelength. A Titan’s atmosphere relevant N_2_/CH_4_ (5%) gas mixture, provided by Air Liquide (purity of 99.999%), is introduced up to 1 mbar total pressure by using a 10 sccm (standard centimeter cube per minute) range flow controller from the underside. This concentration of CH_4_ was chosen because it is known to produce the maximum amount of tholin in plasma discharge experiments^22^. The thickness of the indium diaphragm makes it impossible to work with a pressure differential greater than 10 mbar. In order to ensure a comfortable margin, the working pressure in the reactor has therefore been set at 1 mbar, one order of magnitude below the limit. When optical absorption happens, then the transmission at a specific pressure is given by the Beer–Lambert law:

$$\frac{{I}_{t}}{{I}_{0}}={e}^{-d\times [X]\times {\sigma }_{X}(\lambda )}={e}^{-\tau },$$

where *d* stands for the length of the optical path, [X] the density of the gas and *σ*_*X*_(*λ*) is the absorption cross section at incident wavelength. Then, the optical depth, *τ*, is defined as the product of these three parameters. As an indication, the distance after the indium membrane at which the absorption of the radiation reaches a maximum (*τ* = 1 and a transmission of 37%) in the reactor is around 8 cm (while our reactor is more than 20 cm long), considering that we have a non-homogeneous medium with two different gases present and that each contributes to the extinction. The contribution of each of the gases must then be summed: ${\sum }_{i}\,[{X}_{i}]\times {\sigma }_{{X}_{i}}(\lambda )$. At 89.2 nm, the absorption cross section of N_2_ is 4.17 × 10^−19^ cm^2^ and that of CH_4_ is 5.20 × 10^−17^ cm^2^ (Southwest Research Institute database: https://phidrates.space.swri.edu) and the total gas density is 2.43 × 10^16^ cm^−3^ linked to the working pressure (1 mbar) via the perfect gas law.

**Figure 4:**
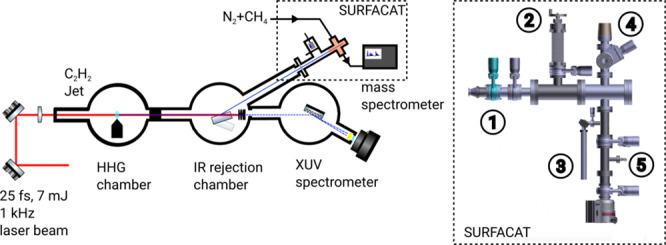
Schematic diagram drawn by the authors of the HHG beamline and the SURFACAT setup: (1) Indium filter, (2) retractable XUV photodiode, (3) cold trap, (4) entrance of the mass spectrometer, (5) gas inlet.

The relatively low working pressure in this work also makes it possible to minimize quenching of excited nitrogen states and to have three-body reactions whose rate is not significant. Pressure affects the density of reactive species (electrons, ions and radicals) and it has been shown that the incorporation of nitrogen into tholins and the degree of aromaticity are pressure-dependent^82^. However, this pressure is not sufficient to reproduce the exact conditions of aerosol production. In Titan, from the temperature and pressure conditions of the thermosphere (700 km), the mean free path is estimated at several tens of centimetres. This value is of the same order as the dimensions of the SURFACAT tholin generation chamber, which is a *ConFlat* (CF)-63 cross piece stainless steel reactor, presented in Fig. 4. Reproducing atmospheric conditions similar to those of Titan would result in a very low collision frequency over excessively long periods of time, which would not produce enough photoproduct for analysis. In this work, an equilibrium is achieved, with a typical mean-free path of *ca*. 0.1 mm which allows us to simulate an appropriate degree of molecular interaction without causing high-pressure effects.

As described in the previous paragraphs, having a relatively low mean free path and an optically thick medium means that the reactive zone is a small volume located behind the indium membrane. This minimizes the wall effects that are the main potential artifact in conventional tholin simulation devices, where aerosols deposit as thin films on the reactor walls^43,83–92^. Catalysis processes lead to complex organic films that have a different morphology than the aerosols that form on Titan. However, it is not certain that these films have a drastically different chemical composition making each device for producing tholins useful to study in order to analyze the complex organic species deposited on Titan’s surface. In conclusion, the SURFACAT chamber is small enough to be easily transportable but large enough to minimise the effects of the walls under operating conditions.

Opposite the membrane, the chamber is closed by a glass window to check the alignment of the light. During the entire irradiation period, this window is darkened. CF flanges are the most commonly used for high vacuum applications and allow a rigorous approach to the possible pollution of the chemistry in the chamber by unwanted oxygen sources. Oxygen is only present on Titan in trace amounts, mainly as CO (0.005%). It is therefore important to minimize the presence of oxygen species (H_2_O, O_2_) in the chamber to avoid unusual oxygen incorporation in the chemistry and production of tholins, if they are to be representative of those produced in Titan’s atmosphere. This represents a real experimental challenge, especially since for experiments carried out at relatively low pressure over a long period of time, the effects of possible micro-leaks will add to the residual pollutants contained on the walls of the chamber. So in addition to building a reactor to limit the effects of walls and leaks, we also implemented a protocol to eliminate as much water vapour as possible from the chamber although it will always be present in trace amounts and will react during photochemical experiments. Prior to the experiment the reactor was baked up to 120 °C during 70 hours. The pressure dwindled to ~10^−6^ mbar. We then tested possible leak by switch off the pumping. After 10 days the pressure was at 3.5 × 10^−5^ mbar. Before EUV irradiation, all CF parts were then again backed up to 120 °C for 9 hours. The pressure is monitored with an absolute capacitance gauge and, before each experiment with the injection of the gas mixture up to 1 mbar, the reactor is pumped down to ~10^−6^ mbar by a primary and a turbo molecular pump located in the lower part, in order to clean out the chamber from residual gas traces. During the photochemistry experiments, removable VAT vacuum valves from each side isolate the reactor and ensure a stable pressure on the order of 1 mbar during few hours of irradiation.

The experiments were carried out at room temperature because it is difficult to maintain the very cold temperatures of Titan’s atmosphere over a long period of time. As a consequence, some compounds that should condense at Titan’s low temperatures (<180 K) will remain volatile and generate a relatively different chemistry^86,93^. It should be noted that temperature also affects heterogeneous chemistry with the adsorption of molecules on the surface of the particles, but also the rate constants of reactions, impacting the nature and abundance of chemical species. However, although temperature is very important for understanding the physico-chemical processes that occur in Titan’s atmosphere, the effects of temperature on the reactivity of molecules, especially the branching ratios, are largely unknown both experimentally and theoretically.

As our chamber contains an optically thick environment at relatively low pressure irradiated by VUV photons, the production of material will be low. In order, to maximize the detection of the products during our experiment, a cryogenic trap held at liquid nitrogen temperature (77 K at atmospheric pressure) was used to capture the photoproducts *in situ* at the end of the irradiation and accumulate the compounds for an efficient *ex situ* analysis using the MS coupled to the setup. The cold trap is positioned as close as possible from the MS and the setup was minimized as possible to increase the density of products. At the end of each experiment, few hours of irradiation, the valve towards the cold trap at liquid nitrogen temperature is opened, enabling to accumulate the condensable gas-phase products during few minutes in the trap. At this temperature molecular nitrogen and most of C_2_-hydrocarbons are not trapped efficiently^75^. Then, the trap is isolated and warmed up to room temperature during half hour and open on the SURFACAT reactor. The volatile products are released under vacuum and mass spectrometry analysis is performed.

### Mass spectrometry diagnostic

Finally, the top side of the cross piece is connected to a mass spectrometer (HIDEN Analytical HPR-20 QIC) to monitor the neutral gaseous products (1% to 0.01% range) during variable irradiation times in the experiment. In the MS, neutral molecules are ionized by a 70 eV electronic ionization and detected with a resolution of 1 atomic mass unit (u) and over a 100 u mass range. Gas sampling is achieved through a metal-bellow tube radially close to the irradiated chamber (*cf*. Fig. 4), also ensuring a relatively low enough pressure (<10^−5^ mbar) in the MS during the sampling. The Fig. 1 displays a mass spectrum when the MS was connected to the chamber under vacuum (*ca*. 10^−6^ mbar).

### Deconvolution of neutral mass spectrum

Upon ionization in a RGA, neutral species tend to undergo ionizing dissociation, leading to the formation of a specific FP for each species. We used these FPs to deconvolve the mass spectra and retrieve the individual contribution of each species present in the reactor following the method described in detail in Gautier *et al*.^64^. This method assumes that the measured mass spectra is a linear combination of each species concentration multiplied by their fragmentation patterns. This is true if the only source of ion in the mass spectrometer is from neutral-electron interaction in the ion source, which is the case in nominal pressure condition for laboratory RGA.

We briefly remind here the principle of this method: A mass spectrum is decomposed (*i.e*. to retrieve the species relative concentrations) into individual species contribution using interior-point least square fitting on the suspected species fragmentation patterns. These fragmentation patterns are obtained from databases such as the National Institute of Standards and Technology (NIST) (http://webbook.nist.gov/), but are highly dependent of the geometry of the ionization source of the instrument for the measurement. This means that typically, the fragments intensity for a given species measured in a lab can vary by up to 50% compared to databases, rendering decomposition doubtful. The method used here allow for compensating this issue by using a Monte-Carlo sampling of the fragmentation patterns, and performing the mass spectra deconvolution not once but several thousands of times with as many different fragmentation pattern. This allows for the retrieval of the probability densities. The retrieved composition is then corrected to account for the ionization cross section of each compounds to ultimately retrieve the mixing ratio of each individual compound detected in the mass spectrum.

## Supplementary information

Supplementary Information.

Supplementary Information 2.
