# Supplementary material for: On an EUV Atmospheric Simulation Chamber to Study the Photochemical Processes of Titan’s Atmosphere
Source: Sci Rep. 2020 Jun 19;10:10009. doi: 10.1038/s41598-020-66950-6 (PMC7305212; doi:10.1038/s41598-020-66950-6)
Supplement: Supplementary file 1 — Supplementary Information. [file 41598_2020_66950_MOESM1_ESM.docx]

Supplementary Information:

**On an EUV Atmospheric Simulation Chamber to Study the Photochemical Processes of Titan’s Atmosphere**

Jérémy Bourgalais^1,*^, Nathalie Carrasco^1^, Ludovic Vettier^1^, Thomas Gautier^1^, Valérie Blanchet^2^, Stéphane Petit^2^, Dominique Descamps^2^, Nikita Fedorov^2^, Romain Delos^2^, and Jérôme Gaudin^2^

^1^LATMOS-IPSL, Université Versailles St-Quentin, CNRS/INSU, Sorbonne Université, UPMC Univ. Paris 06, 11boulevard d’Alembert, 78280 Guyancourt, France.

^2^CELIA, Université de Bordeaux – CNRS – CEA, UMR5107, 351 Cours de la Libération F33405 Talence, France.


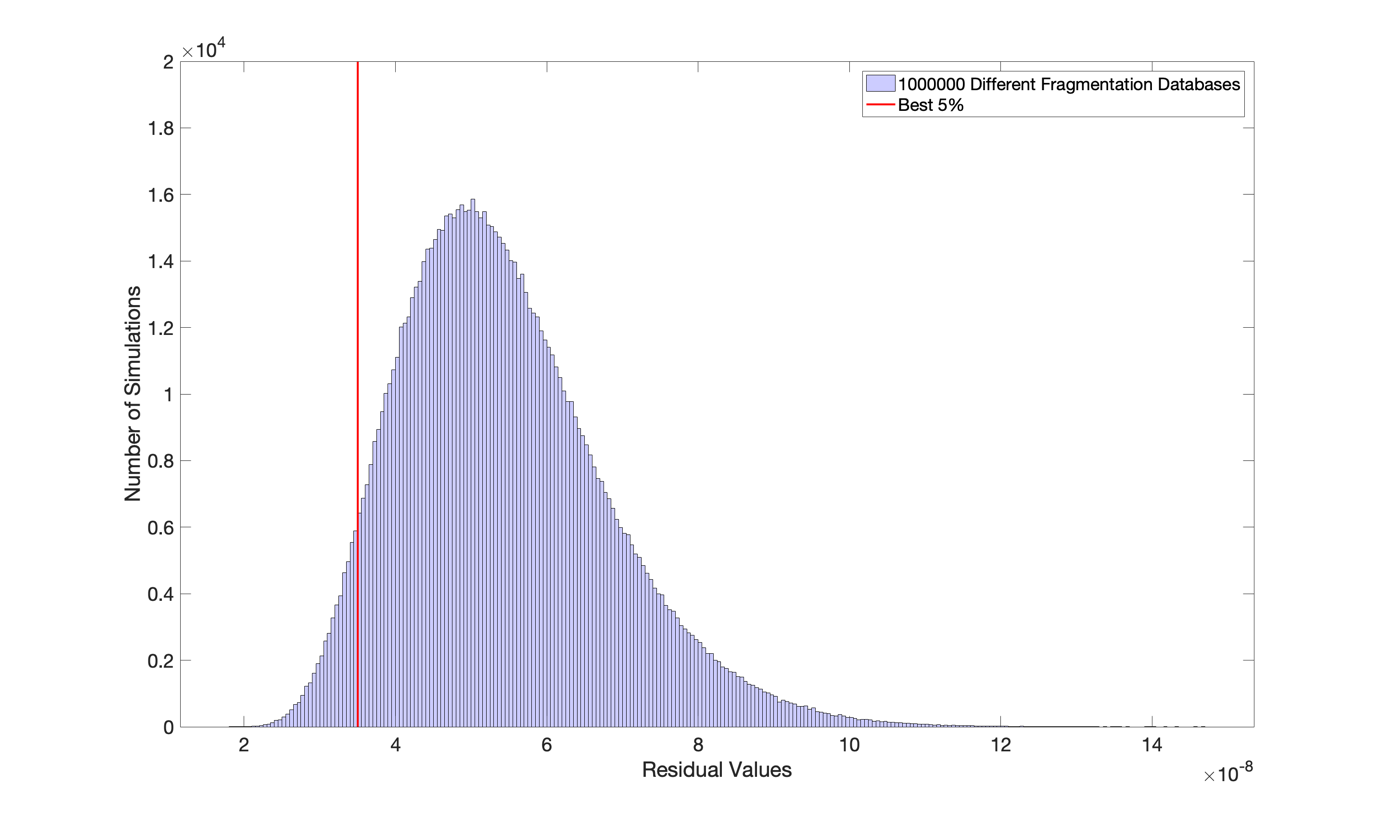


**Figure S1.** The values of the residuals of one million deconvolutions each obtained from a different fragmentation database. The best 5% corresponding to the minimum residue values are kept as a statistical solution.


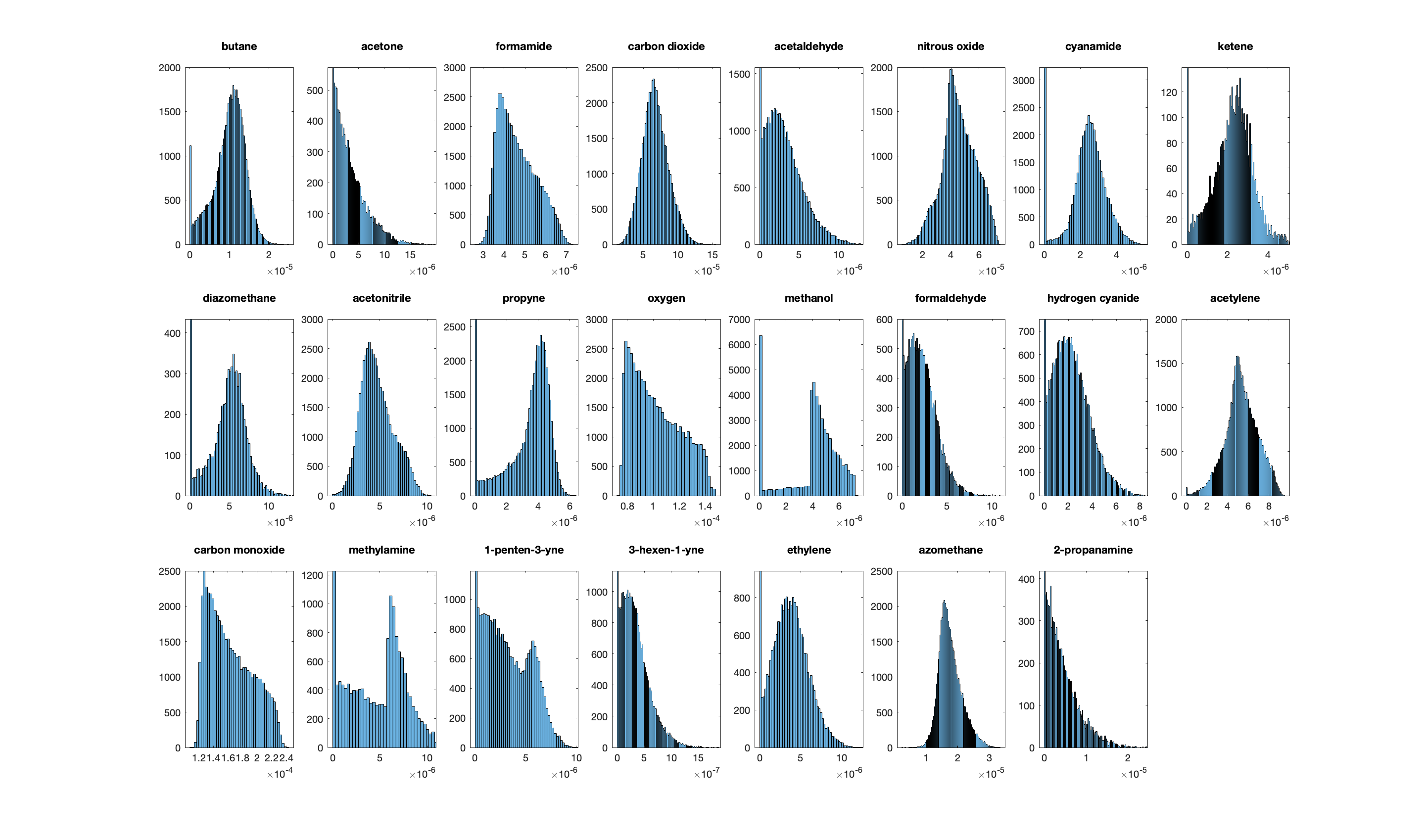


**Figure S2.** Probability densities of the mixing ratio of the molecules in the reactor as retrieved by the Monte-Carlo algorithm.
